# Ion beam processing of DNA origami nanostructures

**DOI:** 10.3762/bjnano.15.20

**Published:** 2024-02-12

**Authors:** Leo Sala, Agnes Zerolová, Violaine Vizcaino, Alain Mery, Alicja Domaracka, Hermann Rothard, Philippe Boduch, Dominik Pinkas, Jaroslav Kocišek

**Affiliations:** 1 Dynamics of Molecules and Clusters Department, J. Heyrovský Institute of Physical Chemistry of the CAS, Dolejškova 3, Prague, 182 23, Czech Republic; 2 Normandie Univ, ENSICAEN, UNICAEN, CEA, CNRS, CIMAP, Boulevard Henri Becquerel, BP 5133, 14070, Caen cedex 5, France; 3 Electron Microscopy Center, Institute of Molecular Genetics of the CAS, Vídenská 1083, 142 20, Prague, Czech Republic

**Keywords:** DNA nanotechnology, DNA origami, FIB, heavy ions

## Abstract

DNA origami nanostructures are emerging as a bottom-up nanopatterning approach. Direct combination of this approach with top-down nanotechnology, such as ion beams, has not been considered because of the soft nature of the DNA material. Here we demonstrate that the shape of 2D DNA origami nanostructures deposited on Si substrates is well preserved upon irradiation by ion beams, modeling ion implantation, lithography, and sputtering conditions. Structural changes in 2D DNA origami nanostructures deposited on Si are analyzed using AFM imaging. The observed effects on DNA origami include structure height decrease or increase upon fast heavy ion irradiation in vacuum and in air, respectively. Slow- and medium-energy heavy ion irradiation results in the cutting of the nanostructures or crater formation with ion-induced damage in the 10 nm range around the primary ion track. In all these cases, the designed shape of the 2D origami nanostructure remains unperturbed. Present stability and nature of damages on DNA origami nanostructures enable fusion of DNA origami advantages such as shape and positioning control into novel ion beam nanofabrication approaches.

## Introduction

Ion beam interaction with DNA origami nanostructures is rarely explored, yet promising applications are foreseen to require such information. DNA nanostructures have been explored as drug delivery vessels for chemotherapeutics [1–2]. With the constant pursuit of effective targeting strategies [3], they could eventually be used in tandem with ion beam therapies against cancer. Another unique application is in long-term data storage [4–5], and ion beams can be used to test the stability of such DNA-origami-based storage under irradiation from natural sources such as cosmic rays or radioisotope decay [6].

More important in the present context are works on the use of DNA origami nanostructures in top-down or bottom-up nanopatterning approaches [7–10]. So far, DNA origami has been proposed only as a resist or as a platform to precisely arrange nanostructure precursors in lithography [11–13]. Incorporating them in procedures based on direct ion beam exposure has so far been avoided because of concerns regarding uncontrollable radiation damage to these soft matter nanostructures. The induction of strand breaks by the direct and indirect effects of ionizing radiation on DNA is a well-known fact [14–15]. However, the situation can be different in DNA origami nanostructures stabilized by highly cross-linked and compact structures. When deposited on the surface, the strong immobilization by cations in between the DNA origami nanostructures and a hydrophilic substrate offer additional stability [16]. Indeed, we have observed the stability of DNA origami nanotriangles in dry and aqueous conditions upon exposure to high doses of ionizing radiation in the low linear energy transfer (LET) regime [17]. These results demonstrate DNA origami’s suitability for fundamental studies with ionizing radiation and now present an opportunity for their use in combination with ion beam processing.

In the present work, we focus on the stability of DNA origami nanostructures deposited on the surface upon irradiation with heavy ions at different interaction regimes that model the most common types of ion processing modalities [18].

The first type of irradiation, at energies above 1 MeV per nucleon, is an example of swift heavy ion (SHI) irradiation. SHI interaction with materials is dominated by electronic stopping power. Electronic excitation typically forms narrow (several nanometers in diameter) ionization tracks in the direction of the primary ion’s initial momentum. Hillock structures are usually formed upon such interaction with single-crystal materials [19], while craters and particle tracks form on polymeric thin films such as PMMA [20–21]. The dimensions of such features can be influenced by the interplay of various factors concerning material properties and the parameters of the impinging ion beam [22–25]. While crucial for modern nanotechnology, SHI cause severe damage to DNA [26–28]. This challenges the use of DNA-based nanomaterials for combined top-down and bottom-up nanoprocessing utilizing heavy ions. However, as previously pointed out, the folded configuration of DNA origami nanostructures offers additional stability against lower-LET ionizing radiation. Could the folded structure of the DNA origami also deal with initial damage around the ion track and conserve its structure in ion beam nanoprocessing?

Heavy ions at medium energies (300 keV to 50 MeV) [29] are still interacting with materials by electronic excitation, but mostly at the beginning of their track. At the end of the track, the ions are predominantly slowed through nuclear stopping. These combined interaction regimes become the basis of commonly used material processing techniques such as high-energy ion implantation, widely applied in laser, detector, and semiconductor industries [30].

Finally, at low (keV) energies, the interaction of heavy ions is dominated by nuclear stopping, which is used in the most common type of beam processing, namely focused ion beam technology (FIB). Following the results of high- and medium-energy ion irradiation on deposited DNA origami nanostructures, which will be presented in this work, we wanted to explore whether we could observe similar effects using a commonly used method for nanofabrication such as FIB, which also happens to cover the low-energy interaction regime. The method is widely available as a complement to scanning electron microscopes. Focused ion beams allow for both subtractive and additive nanoscale manufacturing [31] and can also be used for chemical analysis and imaging [32]. It is also worth mentioning that at high projectile charge states, the ions can be imagined as deep potential energy well, allowing for further surface interaction [33]; however, this will not be explored in the present study.

The ability of ion beams to confine damage to the nanometer scale and the nanometric precision of DNA origami-based assembly open possibilities in more precise tuning and control of nanofabrication. Here we analyze the consequences of ion beam irradiation on 2D DNA origami nanotriangles deposited on Si as a model substrate and resulting nanostructure modifications, which can be further exploited in novel material processing at the nanoscale.

## Experimental

### DNA origami synthesis

Rothemund 2D triangles were synthesized by mixing 6 nM M13mp18 scaffold ssDNA with 144 nM staple strands in 1× TAE buffer supplemented with 12.5 mM MgCl_2_ (folding buffer, FOB) and additional MilliQ water to reach the desired final volume (usually 100 μL for a standard synthesis). We used a higher excess of the staple strands because of the already long storage time of our staple stock solution [34]. The mixture was then annealed to 90 °C and slowly cooled down at a rate of −7 °C·min^−1^ for about 2 h. Afterwards, 100 μL of the synthesis mixture was filtered through 100 kDa MWCO Amicon centrifugal filter units to remove excess staple strands. This was done three times, each time adding 400 μL of FOB, at a relative centrifugal force of 4000*g* for 4 min. The filters were then flipped and spun on clean tubes to recover the purified sample. Typical yields from this procedure are about 50 μL of 12–15 nM DNA origami as estimated from the UV absorbance at 260 nm measured using a Denovix DS-11 FX+ spectrophotometer. These were then diluted to the desired concentration as required.

### Preparation of dry samples and AFM imaging

Silicon wafers were cut into ∼7 × 7 mm^2^ chips and were then plasma-cleaned in air using a Roplass RPS40+ plasma cleaner, which generates a thin layer of plasma by diffuse coplanar surface barrier discharge [35]. The Si surface is exposed to the thin plasma layer for at least 10 s at a distance of 0.2 mm. A volume of 1 μL of a 6 nM DNA origami solution was then dropped onto the Si chips together with 15 μL of 10× FOB and allowed to incubate for 1 h over an ethanol bath. The surfaces were thoroughly washed with at least 1 mL of 50% ethanol and then dried carefully with N_2_. We also performed several depositions on mica, but the material was found to be unsuitable for the irradiation studies at such high fluences because of significant macroscopic ion-induced damage through cracking and flaking of the upper layers after irradiation. Therefore, in the present study, we only evaluated samples on Si.

AFM imaging was used to check and subsequently analyze the irradiated samples. The imaging was performed in air using a Dimension Icon AFM (Bruker) in ScanAsyst mode which employs PeakForce Tapping Technology and ScanAsyst probes (40 kHz, 0.4 N/m). Image processing was limited to flattening the images using the Gwyddion software [36], which was also utilized to generate height profiles of desired regions of interest.

### Heavy ion beam irradiation

The ion beam irradiation experiments were performed at GANIL (Grand Accélérateur National d’Ions Lourds) in Caen, France. Irradiations were carried out in two beamlines: at IRRSUD (IRRadiation Sud) for the lower energy (0.7 MeV/u) and IRABAT (IRradiation À BAsse Temperature) for the higher energy (60 MeV/u). Both beamlines are equipped with a beam sweeping device allowing for uniform irradiation (typical irradiation field: 5 cm × 5 cm; ion fluence accuracy: ∼5%; horizontal frequency: 400 Hz; vertical frequency: 40 Hz) and dedicated dosimetry [37].

At the lower-energy beamline, a 0.7 MeV/u ^56^Fe^10+^ beam was used. Dry samples were adhered onto an aluminum plate using a double-sided carbon tape (which also removes accumulated heat and accumulated charges). The samples were irradiated facing the beam inside a vacuum chamber (10^−8^ mbar) at room temperature. The projectile flux was deduced from the current measured continuously during irradiation on the four slits used to limit and define the irradiation field. Before irradiation of samples, a Faraday cup is inserted and the ratio between the Faraday cup and slit currents is determined and allows for calculating the reached projectile fluence. Fluxes were kept at or below 2 × 10^9^ ions·cm^−2^·s^−1^ to prevent macroscopic sample heating. Projectile fluences ranged from 10^12^ to 10^13^ ions·cm^−2^. Control samples were adhered at the back of the aluminum plate, in a region not exposed to the ion beam.

For the high-energy irradiation, a 60 MeV/u ^56^Fe^25+^ beam was used. Samples were irradiated in air on the outer surface of a cell culture flask oriented to face the beam. Maximum flux was set to 2.5 × 10^8^ ions·cm^−2^·s^−1^. The ion flux was deduced from the measurement of the beam intensity using a detector based on secondary electron emission from a thin Fe foil placed inside the IRABAT vacuum chamber which allows for online monitoring during the irradiation of the samples. This detector was calibrated beforehand using a Faraday cup.

### Focused ion beam irradiation

A Tescan Amber FIB-SEM was used to etch the sample surface. A Ga^+^ focused ion beam was used (*E* = 30 keV, *I* = 10 pA) to draw 10 μm trenches with a nominal depth of 100 nm using single line scan. This resulted in actual widths of 137 ± 3 nm from rim to rim and depths of ∼41 ± 1 nm from the average surface level to the bottom of the trench, as measured ex situ in AFM. The irradiation was done in vacuum (∼10^−7^ mbar) and at ambient temperature. The default configuration of the sample stage is perpendicular to the electron beam of the SEM component; hence, for irradiation, the sample is tilted at 55° through the motorized compucentric stage from the default configuration to be exactly perpendicular to the ion beam.

## Results and Discussion

### Fe beam irradiation in vacuum

**Figure 1:**
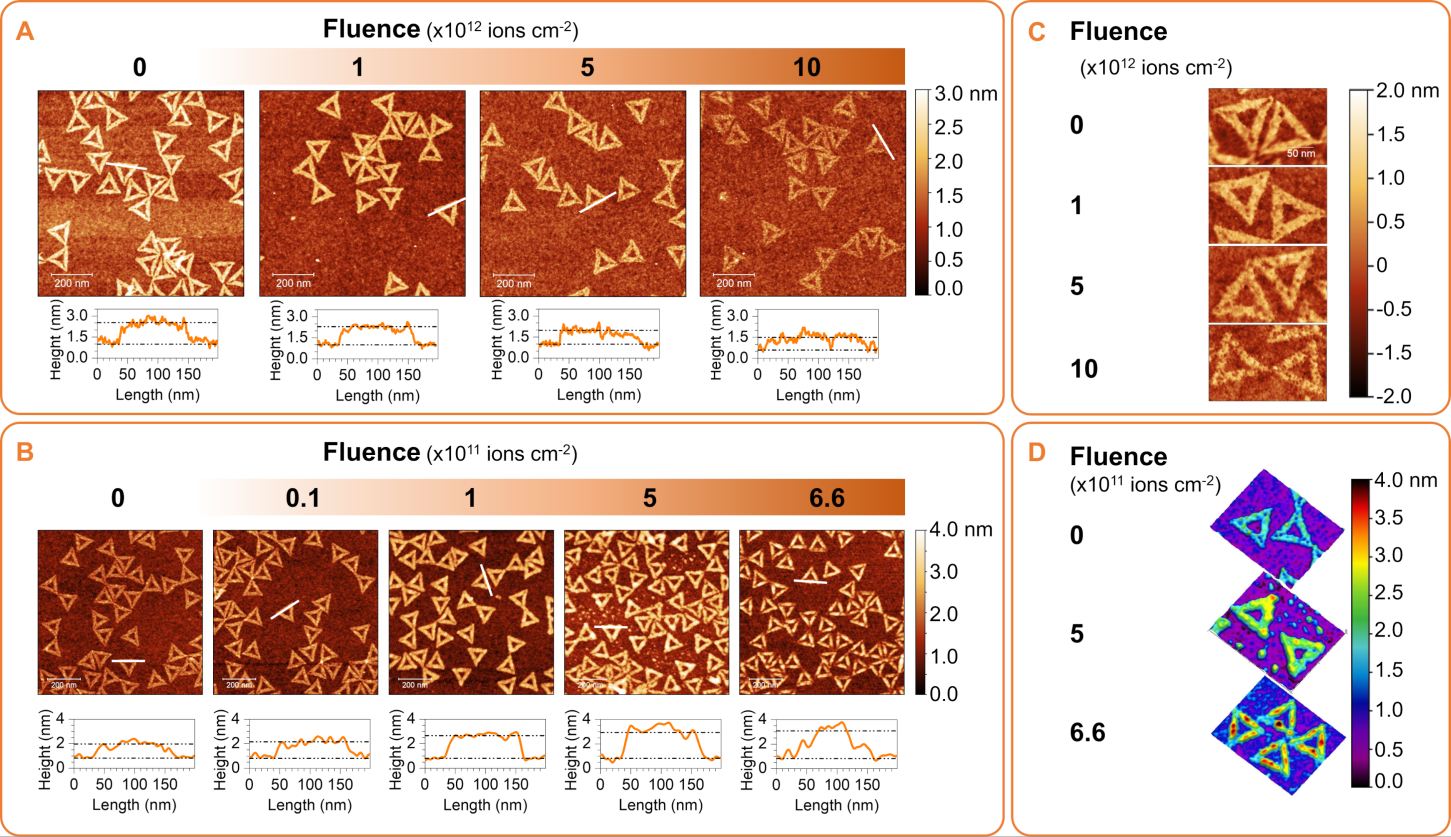
AFM images of DNA origami nanotriangles on Si irradiated with increasing fluences of ^56^Fe^10+^ (0.7 MeV/u) in vacuum (A) and ^56^Fe^25+^ (60 MeV/u) in air (B); the corresponding line profiles of representative trapezoids (white lines) are plotted below each image. Height maps of higher magnification images are shown in panels (C) and (D).

**Figure 2:**
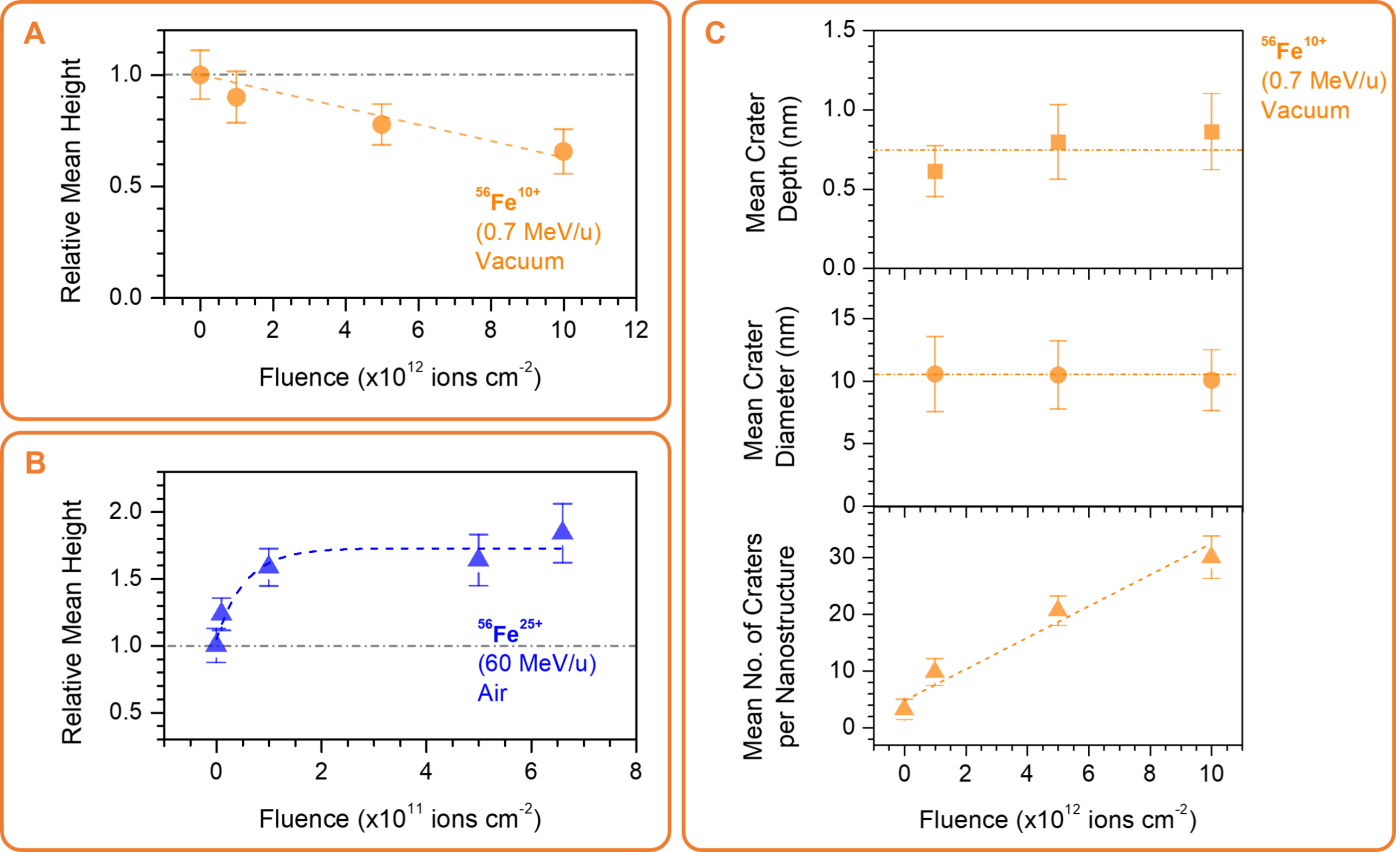
(A) Average height over the area of DNA origami nanotriangles deposited on Si with respect to the control sample upon irradiation with varying fluences of ^56^Fe^10+^ (0.7 MeV/u) in vacuum (error bars reflect the standard deviation among 140–160 triangles profiled from eight AFM images per fluence). (B) Average height over the area of DNA origami nanotriangles deposited on Si relative to the control sample upon irradiation with varying fluences of 56Fe25+ (60 MeV/u) in air. A saturating exponential is approximated as guide for a possible fluence-dependent trend. (C) Estimate of the crater dimensions (extracted from 190–240 craters profiled from seven AFM images per fluence) and the lowest bound for the number of craters formed on DNA origami nanotriangles deposited on Si upon ^56^Fe^10+^ irradiation at various fluences in vacuum. Lines to guide the eye for the figures in (A) and (B) are shown to demonstrate the effect of the fluence.

Dry DNA origami nanotriangles deposited on Si substrates were irradiated with increasing fluences of ^56^Fe^10+^ ions (0.7 MeV/u). The overall triangular shape of the control sample is preserved, but two observable dose-dependent changes occur, namely height loss and formation of craters on the nanostructures. Figure 1A shows AFM images of control and irradiated samples with the height profiles of one side of representative nanotriangles at each fluence level plotted at the bottom of each image. The relative mean height of the nanotriangles in comparison to the unirradiated control sample is plotted in Figure 2A as a function of the fluence. We associate this decrease in height to localized sample heating, which can desorb residual H_2_O and loosely bound DNA radiolysis products especially in vacuum. We performed macroscopic sample heating experiments (Supporting Information File 1, Figure S1), and we detected an observable height loss starting from 150 °C. This is consistent with what has been observed in heating experiments on dry DNA origami in Ar atmosphere [38]. At 250 °C, it has been observed that the nanostructures begin to be pyrolyzed [39], plausibly leaving inorganic residues such as Mg and P [38]. It is worth mentioning that neither 250 °C nor 150 °C was reached under the irradiation conditions in the present experiments on the macroscopic level. The samples are placed on an aluminum block, and even at the highest fluences the substrate temperature is below 50 °C. The observed material evaporation resulting in the loss of nanostructure height must be associated with the energy transfer within the DNA nanostructure or highly localized effects, such as thermal and pressure shock waves in the vicinity of the track [40]. We are now preparing experiments to explore this issue.

The formation of craters on the nanostructures was apparent from a fluence of 1 × 10^12^ ions·cm^−2^. Higher-magnification images of representative triangles are shown in Figure 1A, and the crater features from the height profiles are described in Figure 2C. The latter was estimated from 1D line profiles traversing the identified craters similar to the illustration shown in Supporting Information File 1, Figure S2. There is an almost linear increase in the number of craters with fluence, but the average crater features remain the same. The mean crater diameter is about 10 ± 3 nm with a mean depth of 0.8 ± 0.2 nm. We associate this to the particle tracks created by the ^56^Fe^10+^ ions. In experiments where the surface was fully covered, such as in the work of Thomaz et al. on 2 nm thick PMMA films on Si irradiated with 1.1 GeV Au atoms (∼5.6 MeV/u) [41], the number of incident ions to the number of craters is 1:1. We expect the same behavior in the present case; however, the evaluation of the crater to fluence ratio is not straightforward. In our case, the craters in the DNA nanotriangle structure can be evaluated only if they lie fully inside the nanostructure. Also with this sample, we can only confidently probe 14 to 18 nm widths of the top surface of the trapezoids, and given that the crater sizes are about 10 nm, the number of craters that can be unambiguously identified is low. Therefore, we can estimate only the lowest bound for the number of craters at the given incident ion fluence, which is depicted in Figure 2C.

### High-energy Fe beam irradiation in air

The higher-energy irradiation ^56^Fe^25+^ (60 MeV/u) could only be performed in air and at lower fluences to avoid heating of the sample due to high energy transfer to the substrate as well as to avoid activation of irradiated components. Figure 1B shows AFM images of samples irradiated with increasing fluences of ^56^Fe^25+^ ions with height profiles of representative triangles shown at the bottom of each image. A height map at higher magnification is also presented in Figure 1D, and the relative height increase is plotted as a function of the fluence in Figure 2B. In this irradiation experiment, craters are not evident, but the sample height increases significantly with the dose. Some height gain and bloating had been observed in UV-irradiated DNA origami nanostructures at low doses [42]. In this previous work, although the samples were irradiated in solution, the AFM analysis was done in the dry state. The gain in height and lateral dimensions at low doses was associated to the expansion of the origami nanostructures due to gradual nicking or strand-breaking rendering the structures more loose [42]. In dry irradiations, the generated broken ends from strand breaking have restricted lateral movement, which probably pushes them to be only lifted-off leading to an observed increase in height. Because the irradiation was performed in air, various reactive species can be also generated, which can change the microenvironment around the nanostructures. This can be influenced by ambient humidity and the generation of plasma from air affecting the nanostructures and DNA–substrate interactions. Despite the underlying mechanism, which is worth of further exploration, this behavior should be emphasized in the present content. Random irradiation of the origami-deposited nanostructures results in a uniform change in the DNA origami shape, which can be additionally controlled by the nanostructure design. Particularly, we can see that the height increase occurs mostly on the central seams of the trapezoids of the DNA origami. The DNA origami design can be used to control not only the shape of DNA origami but also their transformation upon irradiation.

### FIB processing

**Figure 3:**
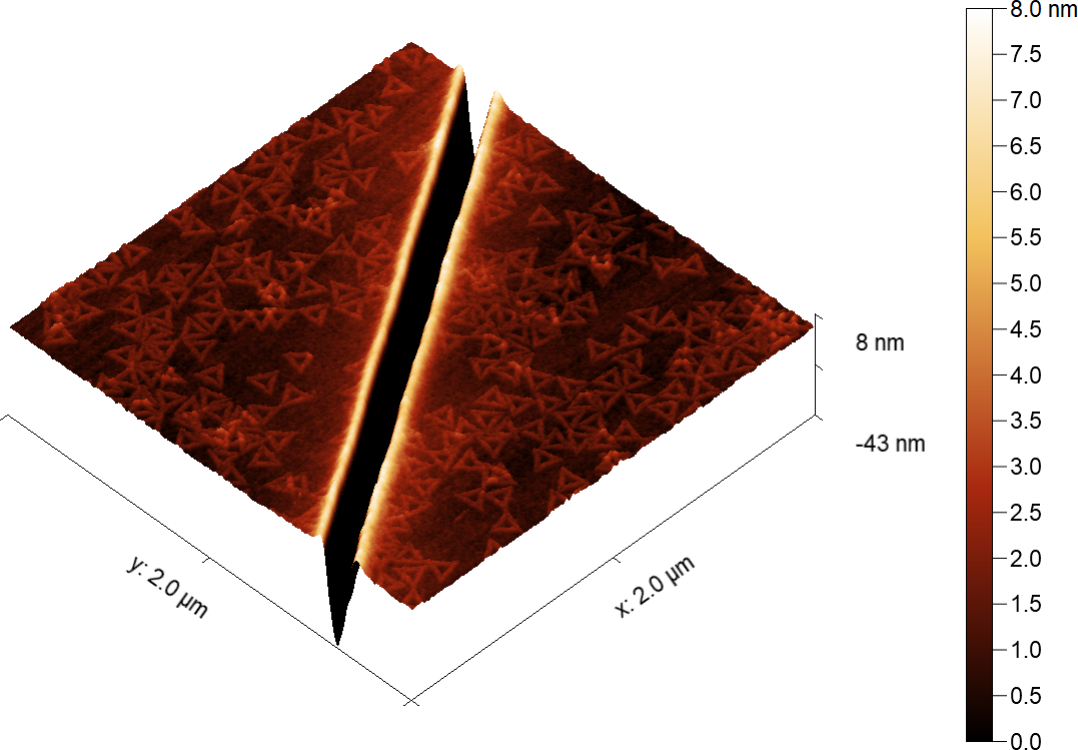
AFM image of a Si surface deposited with DNA origami nanotriangles and then etched with FIB (Ga^+^, 30 keV, 10 pA). The height gradient scale is shown on the right; its range is chosen to reveal the surface features and the deposited DNA origami nanostructures. Everything below 0 nm appears black (i.e., the bottom of the trenches).

Inspired by the shape preservation of DNA origami nanostructures under ion beam irradiation, we explored the damage response under a conventional focused ion beam typically used in lithography. Lines of about ∼140 nm in width ∼40 nm in depth were drawn on the surface using a FIB-SEM. Although the lines could be clearly observed, the nanostructures could not be resolved in FIB or SEM imaging modes because of poor contrast for both and the observed sample charging during SEM imaging (Supporting Information File 1, Figure S4). Because of this, we reverted to AFM to check on the sample damage. An AFM scan of one of the etched areas is shown in Figure 3. The nanostructures around the FIB-etched line remain intact. A ∼8 nm high rim is also formed, which is typical for ion irradiation due to the melt flow pushing some material outward of the trenches [21]. Even in these elevated areas very close to the line edge, the trimmed DNA origami nanostructures merely follow the contours. For the FIB-SEM setup used, this was the narrowest beam generated. Reducing the beam size would allow for even more precise trimming of the nanostructures for lithographic applications.

## Conclusion

**Figure 4:**
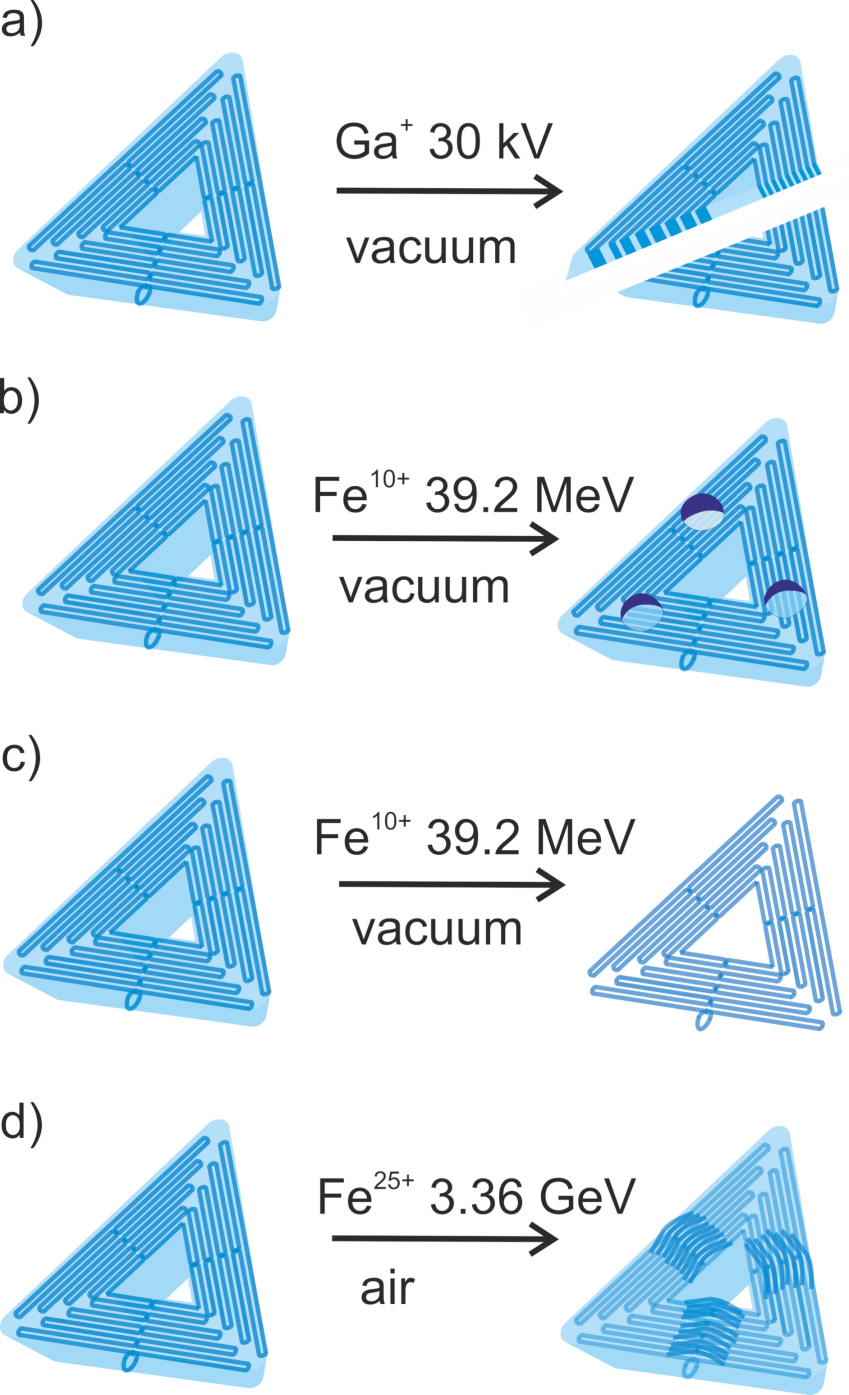
Artistic representation of four types of effects that can arise from ion beam interactions with DNA origami nanostructures on Si as reported in present work. (A) and (B) are shaping effects via focused beam milling or crater formation. (C) and (D) are height changes induced in vacuum and in air, respectively. In vacuum, material from the structures may sublime. In air, it is plausible that the stochastic irradiation transforms into uniform changes on the structure, which can be controlled by DNA origami design.

We explored model ion interactions with DNA origami nanostructures, showing promise for fusing these state-of-the-art nanotechnology approaches. The main effects of ion beams on nanostructures are shown in Figure 4A–D. The most important observation is that the shape of the folded DNA nanostructures deposited on the surface remains conserved even upon significant energy input. The ions at low and medium energies can be used to shape the nanostructures via trimming, as demonstrated using FIB, or via crater formation, as demonstrated using ^56^Fe^25+^ (0.7 MeV/u) ion impact. Such shape modifications are becoming important with the development of higher-order DNA origami nanostructures [43], particularly lattices for surface nanopatterning [44–45]. Ion beam lattice trimming can be used in combined lithographic approaches as well as in forming well-defined nano-bio interfaces. While we demonstrate here that the trimming of individual DNA nanostructures within the lattice is possible, the collective response of the lattice and defect formation as a response to ion impact represents an interesting direction for future studies.

The processes leading to height modification of nanostructures in Figure 4C,D are interesting from the perspective of energy transfer when random highly localized ionizing radiation events are transformed into uniform effects over the nanostructures. The desorption of presumably organic material from the nanostructures leaving heavy atoms on the surface organized on the DNA origami pattern can be used as an alternative technique to already existing DNA-based methods for metallic nanostructure preparations [46]. DNA origami can be loaded with various metal ions [11,47–48]. Additionally, nanostructures modified by metal coverage [49] or nanoparticle binding [50–51] can be used to bring metallic materials to the surface at unprecedented resolution. Irradiation of such metal–DNA origami nanostructures with ion beams can be used to manufacture metallic nanostructures with sub-nanometer resolution.

Finally, the localized DNA origami height variations in Figure 4D could represent a unique link between the DNA origami design and the ion beam modification. The results of height profiling, however, indicate no drastic changes in the microenvironment of the DNA origami nanostructures on the substrate upon irradiation. This might not be the case, as seen, for ion beam irradiation in air. The height profiles may also be sensitive to environmental conditions especially the nature and availability of counterions [52]; hence, there is a need for in situ chemical analysis to fundamentally explore these effects, which are, at the moment, complicated to install in large infrastructures. Nonetheless, it is possible that the most probable mechanism of the height variation could be the radical attack from the environment when the irradiation is done in air. A similar behavior was observed also upon chemical modification of the nanostructures [38]. DNA origami could be therefore designed in a way that a specific segment of the origami is modified/lifted upon stochastic energy deposition or radical attack, resulting in uniform modifications of the nanostructures.

## Supporting Information

Height evolution upon thermal processing of DNA origami deposited on Si, example of crater measurements from line profiles on AFM images, variation of surface coverage with ion beam fluence, and SEM and AFM images of trenches etched by FIB on DNA-origami-covered Si.

File 1 Additional experimental data

## Data Availability

All data that supports the findings of this study is available in the published article and/or the supporting information to this article.
