# Supplementary material for: Ion beam processing of DNA origami nanostructures
Source: Beilstein J Nanotechnol. 2024 Feb 12;15:207–14. doi: 10.3762/bjnano.15.20 (PMC10877082; doi:10.3762/bjnano.15.20)
Supplement: File 1 — Additional experimental data [file Beilstein_J_Nanotechnol-15-207-s001.pdf]

BEILSTEIN JOURNAL OF NANOTECHNOLOGY

Supporting Information

for

Ion beam processing of DNA origami nanostructures

Leo Sala, Agnes Zerolová, Violaine Vizcaino, Alain Mery, Alicja Domaracka, Hermann Rothard, Philippe Boduch, Dominik Pinkas and Jaroslav Kočišek

Beilstein J. Nanotechnol. **2024,** *15,* 207–214. doi:10.3762/bjnano.15.20

Additional experimental data

License and Terms: This is a supporting information file under the terms of the Creative Commons Attribution License (https://creativecommons.org/licenses/by/4.0). Please note that the reuse, redistribution and reproduction in particular requires that the author(s) and source are credited and that individual graphics may be subject to special legal provisions.
The license is subject to the *Beilstein Journal of Nanotechnology* terms and conditions: (https://www.beilstein-journals.org/bjnano/terms)

List of Figures

Temperature dependence of DNA origami height S2

Estimation of crater dimensions from an AFM image S3

Variation of surface coverage with ion beam fluence S3

SEM and AFM images of trenches etched by FIB on DNA-origami-covered Si S4

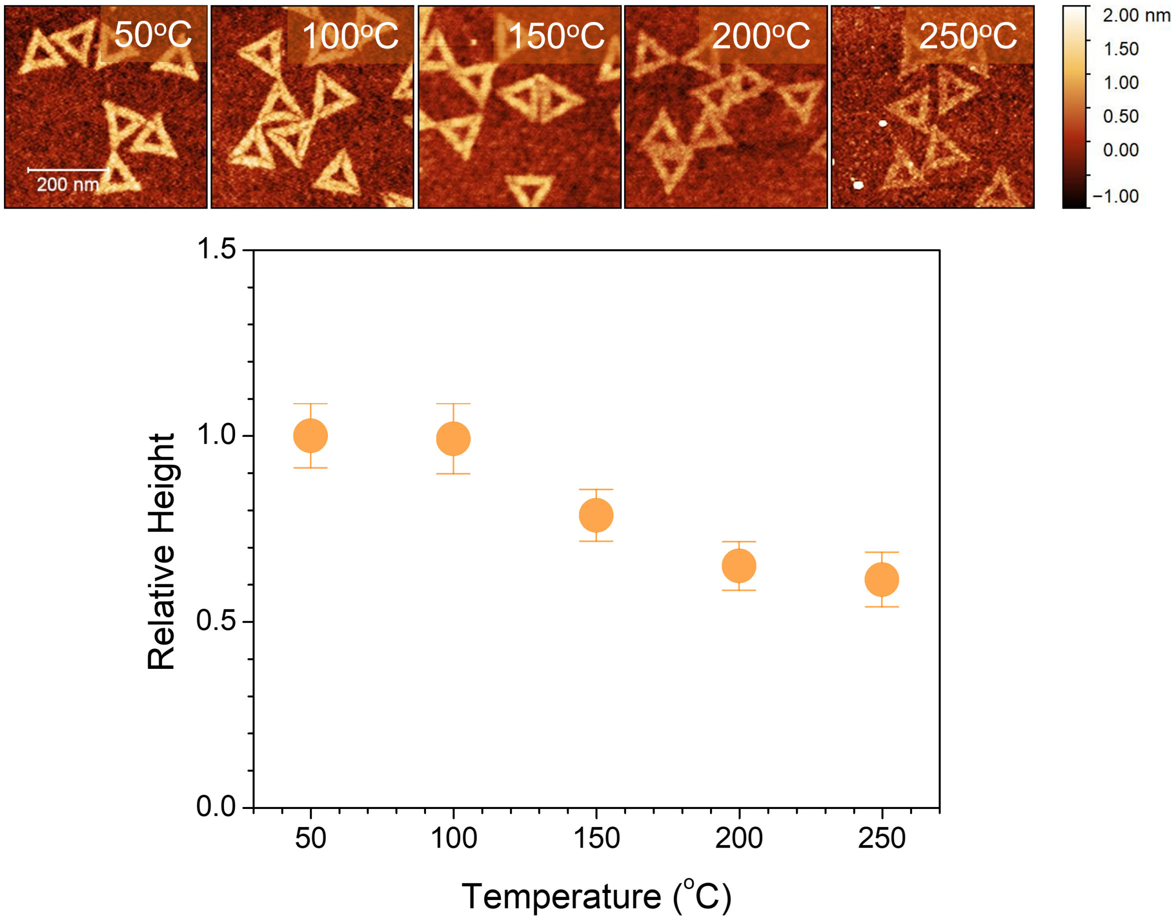

Figure S1: Relative height loss (extracted from AFM images) of DNA origami nanotriangles deposited on Si upon thermal treatment in vacuum.

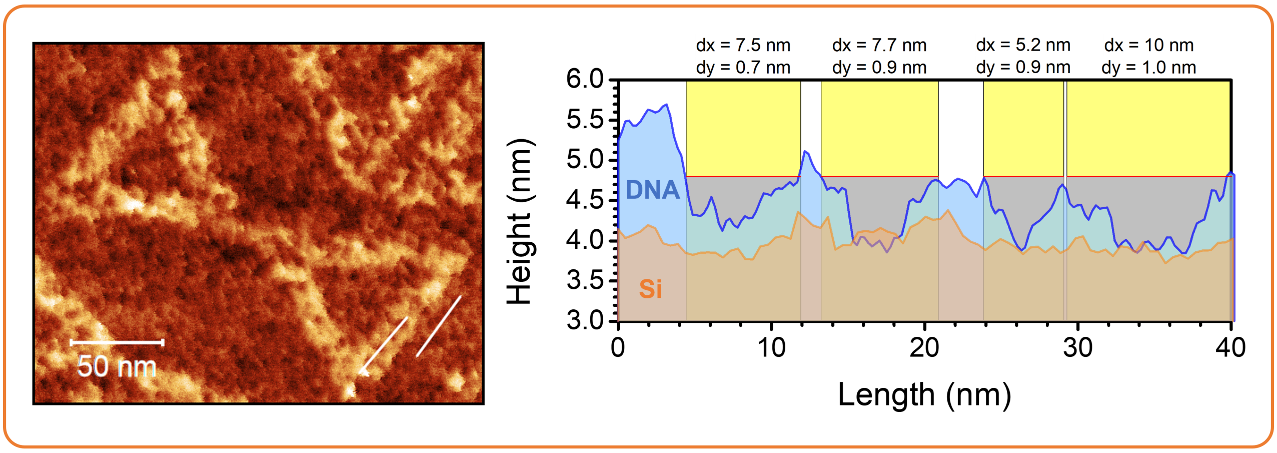

Figure S2: Example of the estimation of crater dimensions from AFM images. Shown here is a high resolution image of DNA origami nanotriangles on Si irradiated with 1×10^{13} $^{56}Fe^{10+}$ions cm^{-2}. The 1D profiles along the white lines (one drawn on the DNA origami nanostructure and another on the Si substrate) are plotted on the left panel with estimated crater diameter (dx) and crater depth (dy) highlighted for each identified crater.

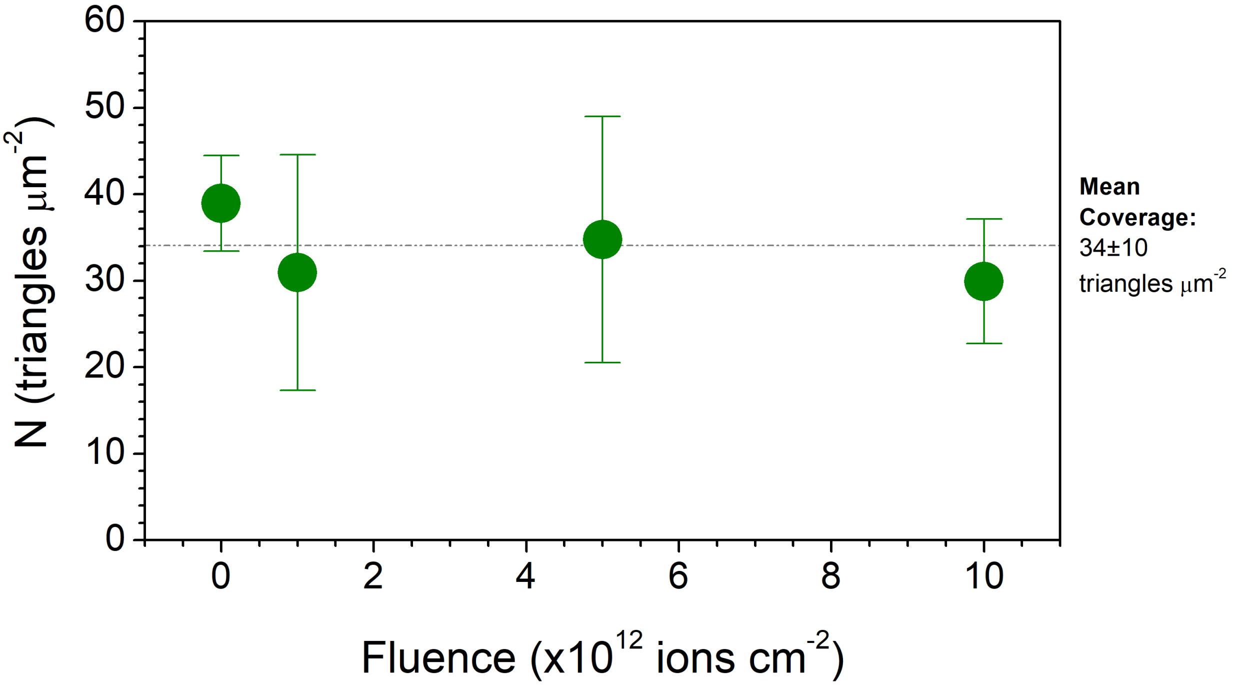

Figure S3: Surface coverage (N) in terms of the number of DNA origami nanotriangles in a 1 μm^2 area of samples irradiated with different fluences of $^{56}Fe^{10+}$ions in vacuum.

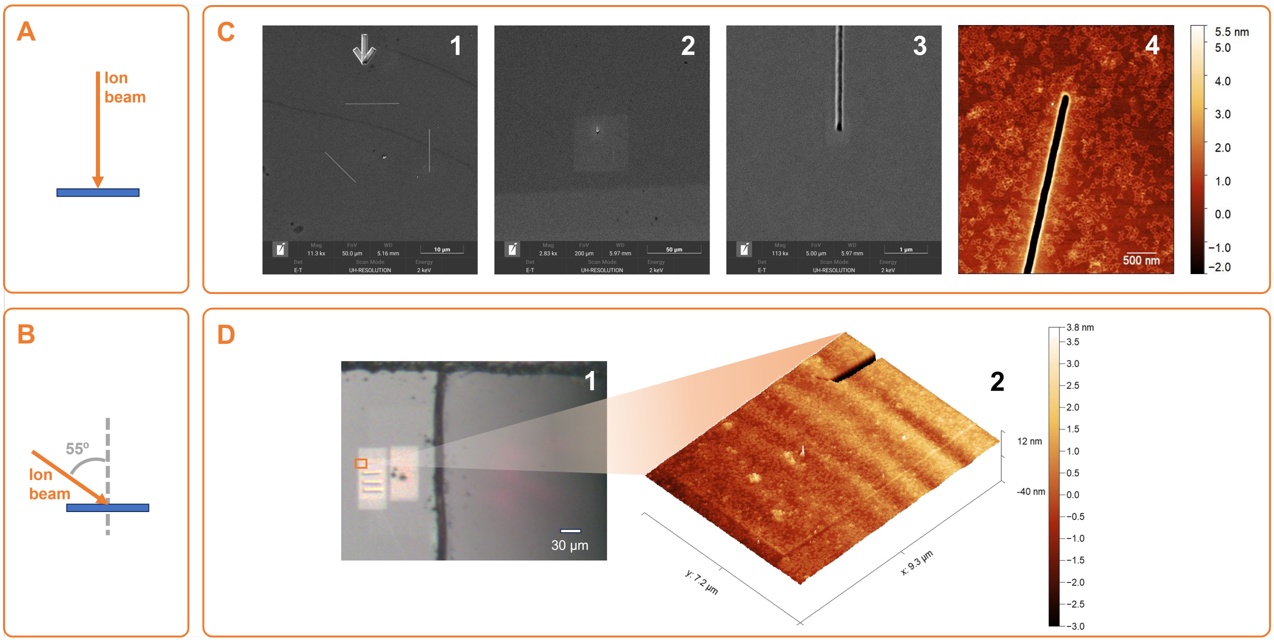

Figure S4: Representative SEM (2 keV beam) and AFM images of FIB-etched lines over DNA-origami-deposited Si at irradiation configuration illustrated in panel A are shown in panel C. Image C1 shows an 11.3 kx magnification (scale bar: 10 μm) focusing on one of the areas etched by FIB. Image C2 is a lower magnification (2.8 kx, scale bar: 50 μm) SEM image of the area in C1 and shows sample charging from the previous imaging on higher magnification. Image C3 is a zoom (113 kx magnification, scale bar: 1 μm) into one of the etched lines but the DNA could not be clearly visualized due to poor contrast and possible sample charging. Image C4 (scale bar: 500 nm) is an ex situ AFM image of one of the lines in C1 showing that DNA origami nanostructures are still present on the Si surface up to the borders of the lines/trenches even after FIB processing. We also performed irradiation at an angle as illustrated in panel B. The representative optical microscope image is shown in image D1 (scale bar: 30 μm) and the corresponding AFM profiling of the region within the orange box is shown in D2 (7.2 x 9.3 μm^2). The SEM-exposed regions are still visible in both images, but they show minimal damage to the exposed DNA origami nanostructures.
